# Effects of Starvation on Growth Characteristics, Locomotor Performance, Foraging Behavior, and Hypoxia Tolerance in Chinese Giant Salamander (*Andrias davidianus*) Larvae

**DOI:** 10.3390/ani16121801

**Published:** 2026-06-11

**Authors:** Jiahong Rao, Zonglin Liu, Shijian Fu, Xiuming Li

**Affiliations:** Laboratory of Evolutionary Physiology and Behavior, Chongqing Key Laboratory of Conservation and Utilization of Freshwater Fishes, Animal Biology Key Laboratory of Chongqing Education Commission of China, Chongqing Normal University, Chongqing 401331, China

**Keywords:** starvation, growth, locomotor performance, foraging behavior, hypoxia tolerance, *Andrias davidianus*

## Abstract

Starvation is one of the common survival stresses frequently encountered by wildlife, and the adaptive strategies of animals to starvation stress exhibit significant interspecific differences. This study investigated the effects of different starvation periods (1–4 weeks) on the growth characteristics, locomotor performance, foraging behavior, and hypoxia tolerance of Chinese giant salamander (*Andrias davidianus*) larvae (5.41 ± 0.06 g; 9.78 ± 0.03 cm). Our results showed that the final body weight of *A. davidianus* larvae decreased significantly with prolonged starvation time, with a more pronounced rate of decrease during the early starvation period. However, the final body length of *A. davidianus* larvae was less significantly affected by starvation stress. Starvation had no significant effects on the relative induction flow speed, relative burst swimming speed, total foraging distance, relative foraging speed, and foraging time of *A. davidianus* larvae. The resting metabolic rate of *A. davidianus* larvae decreased significantly as the starvation period lengthened. Starvation lasting 2–4 weeks significantly increased the dissolved oxygen level at which *A. davidianus* larvae exhibited the loss of equilibrium (ventral side facing upward). These results indicate that (1) starvation has a more significant negative impact on body mass than on body length in *A. davidianus* larvae; (2) starvation stress does not significantly affect the locomotor performance or foraging behavior of *A. davidianus* larvae, but it does lead to a significant reduction in their extreme hypoxia tolerance.

## Simple Summary

Animals adopt diverse morphological, physiological, and behavioral strategies to cope with starvation stress, which can occur at various life history stages. The Chinese giant salamander (*Andrias davidianus*) is the largest extant amphibian species in the world, and its wild population is declining due to habitat destruction and other factors. This study aimed to investigate the effects of starvation stress for 1–4 weeks on the growth characteristics, locomotor performance, foraging behavior, and hypoxia tolerance of *A. davidianus* larvae. The results showed that the locomotor performance and foraging behavior of *A. davidianus* larvae were not significantly affected by starvation stress, which may be beneficial for maintaining normal foraging and predator avoidance activities during starvation. However, starvation led to a significant reduction in body weight and ultimate hypoxia tolerance. Therefore, starvation tolerance time and hypoxia tolerance warrant full attention in the conservation practice and research involving *A. davidianus* larvae.

## 1. Introduction

Due to the uneven distribution of food resources in both temporal and spatial dimensions in nature, animals are frequently subjected to the adverse stress of starvation during their life cycles [1,2,3]. Throughout long-term adaptive evolution, different animals have adopted varied strategies in response to starvation stress [2,4]. Previous studies have found that amphibians typically cope with environmental stress from food shortages by employing physiological or behavioral strategies, such as downregulating daily maintenance energy expenditure and reducing the frequency of daily activities [3,4,5,6]. Due to limitations in food supply, amphibians continuously consume their intrinsic energy stores, and the functions of various organs are subsequently downregulated. Consequently, the body mass of amphibians also decreases significantly with prolonged starvation [7,8,9].

Locomotor performance is a fundamental guarantee for the survival and reproduction of aquatic animals and is significantly influenced by various environmental factors [10,11,12,13,14]. Induced flow speed refers to the water velocity at which an aquatic animal initiates a swimming behavior response and is a commonly used parameter for studying its locomotor characteristics [15,16]. Burst swimming speed reflects the maximum swimming speed an aquatic animal can achieve in a short period and serves as an important parameter for evaluating anaerobic locomotor capacity in aquatic animals [17,18]. Previous studies have found that starvation stress can significantly affect the locomotor performance of aquatic animals. However, due to species-specific differences in tolerance to starvation, the responses of locomotor performance to starvation stress also exhibit significant interspecific variation among different aquatic animals [19,20,21].

Foraging behavior is one of the fundamental life activities of aquatic animals, providing them with nutrients and energy for survival [22,23]. Researchers employ various indicators to examine animal foraging behavior, such as foraging time, food preference, percentage of time spent moving during foraging activity, number of movements per minute, and number of successful prey captures [24,25,26]. The foraging behavior of aquatic animals is influenced by various external environmental factors (e.g., light, temperature, dissolved oxygen) and internal factors inherent to the animal itself (e.g., body size and health status) [27,28,29,30,31,32]. Previous studies have found that amphibians also exhibit different foraging behavior characteristics during starvation, which are closely related to their species and the duration of starvation [33,34].

Dissolved oxygen in water is a crucial environmental factor affecting the survival of aquatic animals [35,36]. Aquatic surface respiration (*ASR*) and loss of equilibrium (*LOE*) are important indicators used to assess the hypoxia tolerance of aquatic animals [36,37]. *ASR* refers to the behavior where aquatic animals ascend to the water surface to ingest atmospheric air to meet their oxygen requirements when the ambient dissolved oxygen falls below a certain threshold, representing their critical tolerance to hypoxic stress. *LOE* refers to the point at which aquatic animals cannot maintain bodily equilibrium (ventral or lateral side facing upward) when the dissolved oxygen drops to a certain level, representing their ultimate tolerance to hypoxic stress [38,39,40]. The hypoxia tolerance of amphibians is influenced by various environmental factors such as altitude (vertical height above mean sea level) and temperature [41,42,43,44]. Previous studies have found that an animal’s metabolic rate is closely related to its hypoxia tolerance [45,46]. Due to their lower oxygen demand, animals with generally lower metabolic rates tend to possess greater hypoxia tolerance [47]. Starvation leads to a decrease in maintenance metabolic energy expenditure in some aquatic animals, thereby positively influencing their hypoxia tolerance [45]. However, other studies have found that starvation can also have a significant negative impact on the hypoxia tolerance of aquatic animals [48].

The Chinese giant salamander (*Andrias davidianus*) (Amphibia: Caudata) is the largest extant amphibian species in the world and is a unique State Key Protected Wildlife species in China [49,50]. Due to overexploitation by humans and the gradual loss of its habitat, the trend of population decline in wild *A. davidianus* is becoming increasingly evident [50,51]. Previous studies have found that the species possesses a remarkable ability to withstand starvation, capable of surviving for dozens of days or even up to a year without food [52,53,54]. To date, research on *A. davidianus* during starvation has involved aspects such as blood parameters [55], intestinal microbiota [56], body composition [57], immune responses [58], and proteomics [59]. However, these studies on the effects of starvation in *A. davidianus* have mainly used subadults or adults as study subjects, while research on larvae remains relatively scarce. Our previous research found that the swimming performance of juvenile *A. davidianus* is not sensitive to meal sizes [60], and long-term fasting leads to a significant decrease in their maintenance metabolism [4]. However, studies on the locomotor function, behavioral traits, and environmental tolerance of the species under starvation stress have not yet been reported in the literature. Although long-term starvation has negative effects on the body weight and certain physiological functions of *A. davidianus*, moderate starvation (starved for 6 days followed by refeeding for 6 days) can increase its feeding rate and digestive enzyme activity, thereby improving its growth rate [61]. Studies on other animals have also found that moderate starvation may positively affect their immune response and lifespan [62,63]. Therefore, this study used *A. davidianus* larvae as the research animal to investigate the effects of 4 weeks of starvation stress on their growth characteristics (body mass and body length), swimming performance (induced flow speed and burst swimming speed), foraging behavior traits (total foraging distance, relative foraging speed, foraging time), and hypoxia tolerance (*ASR* and *LOE*). The aim was to explore their physiological and behavioral adaptation strategies in response to starvation stress, thereby providing theoretical support and foundational data for the conservation practices of this species.

## 2. Materials and Methods

### 2.1. Source of Experimental Animals and Acclimation Conditions

The *A. davidianus* larvae used in this study were purchased from the “Zhujiangyuan” Chinese Giant Salamander Farm in Linwu County (25°10′–25°35′ N, 112°20′–112°47′ E), Hunan Province, China. They were acclimated for 14 days in a recirculating aquaculture system (length × width × height: 180 cm × 50 cm × 50 cm; water depth: 10 cm) at the Laboratory of Evolutionary Physiology and Behavior, Chongqing Normal University. During the acclimation period, the photoperiod, dissolved oxygen level, and water temperature were set at 14L:10D, (7.5 ± 0.5) mg L^−1^, and (20.0 ± 0.5) °C, respectively. The larvae were fed daily at 19:00 with chironomid larvae until satiation. To maintain water quality in the aquaculture system, any remaining feed and feces were removed 2 h after feeding. One-third of the aquaculture water was replaced daily with fully aerated tap water. The pH, GH, and TDS of the aquaculture water were 6.94, 105.50 mg L^−1^, and 226.52 mg L^−1^, respectively.

### 2.2. Starvation Treatment

After 14 days of acclimation, 216 larvae (<1 year old) of similar size (5.41 ± 0.06 g; 9.78 ± 0.03 cm) and good health were selected and randomly divided into a control group (S0) and three starvation groups: 1-week (S1), 2-week (S2), and 4-week (S4) starvation, with 54 individuals per group. In this study, the 216 experimental individuals were measured for body length and weight one by one, and then sequentially randomly assigned to three starvation groups and a control group. The sample size of each group was determined based on the number of samples required for measuring the parameters in this experiment. Each individual was housed in a separate cage (length × width × height: 10 cm × 10 cm × 10 cm) made of wire mesh for the duration of the starvation treatment. The cage dimensions did not prevent the experimental animals from fully turning around or engaging in normal activities. None of the experimental individuals died during the starvation treatment. After the experiment, all experimental individuals were able to feed voluntarily and gradually increased in body weight. Other environmental conditions during the starvation period remained consistent with those during the acclimation period.

### 2.3. Parameter Measurement and Calculation

Body weight and length of the S0 group were measured before starvation. For the S1, S2, and S4 groups, body weight and length were measured once before starvation, and again after starvation when the measurement of relevant parameters was completed. In this study, the body length of the experimental individuals refers to the total length from the tip of the snout to the end of the tail. Parameters related to locomotor performance, foraging behavior, resting metabolic rate, and hypoxia tolerance were measured for the S0, S1, S2, and S4 groups after 0, 1, 2, and 4 weeks of starvation, respectively. The samples used to measure each parameter were randomly selected from the corresponding treatment groups. In this study, we did not exclude any experimental individuals or their data points. During the different stages of the experiment, only the first author was aware of the group allocation. All other investigators were blinded during the allocation, the conduct of the experiment, the outcome assessment, and the data analysis.

#### 2.3.1. Body Mass and Body Length

The body mass and body length of each individual (mildly anesthetized with 50 mg L^−1^ MS-222, tricaine methane sulfonate) were measured before and after the starvation treatment, and the relevant parameters were calculated using the following formulas.

Condition factor (100 × g cm^−3^) = 100 × body mass (g)/body length (cm)^3^ (1)

Daily rate of body mass change (% d^−1^) = 100 × [Final body mass (g) − Initial body mass (g)]/Starvation duration (d) (2)

Daily rate of body length change (% d^−1^) = 100 × [Final body length (cm) − Initial body length (cm)]/Starvation duration (d) (3)

#### 2.3.2. Locomotor Performance

Following the different starvation periods, 10 individuals were randomly selected from each group for the measurement of absolute induction velocity and burst swimming speed. The measurement method is briefly described as follows [60]: After the starvation treatment, each individual was transferred separately into an “Aquatic Animal Swimming Respirometer” (3.5 L) [64] and allowed to acclimate for 1 h with a water velocity set at 5 cm s^−1^ (approximately 0.5 body lengths per second, bl s^−1^). Subsequently, their induction velocity and burst swimming speed were measured. The water velocity in the respirometer was controlled by a connected computer program. After the acclimation period, the water velocity was increased continuously at an acceleration rate of 0.167 cm (s^2^)^−1^. When the individual exhibited a swimming posture characterized by lifting its body off the bottom, holding its limbs close to its sides, and swinging its tail laterally, the corresponding water velocity was recorded as its absolute induction velocity (cm s^−1^). The relative induction velocity (bl s^−1^) was calculated by dividing the absolute induction velocity by the individual’s body length. As the water velocity continued to increase, the individual eventually reached an exhausted state, remaining against the mesh screen at the end of the swimming chamber for more than 20 s without further swimming. The water velocity at this point was recorded as its absolute burst swimming speed (cm s^−1^). The relative burst swimming speed (bl s^−1^) was calculated by dividing the absolute burst swimming speed by the individual’s body length.

#### 2.3.3. Foraging Behavior

Following the different starvation periods, 10 individuals were randomly selected from each group for the measurement of foraging behavior characteristics. The measurement method was as follows: A cylinder (PVC pipe; diameter: 15 cm) was placed on the right side of a custom-built rectangular filming apparatus for feeding behavior (PVC sheets; length × width × height: 120 cm × 20 cm × 20 cm; water depth: 10 cm) to allow the individuals to acclimate to the test environment. Fifty chironomid larvae were placed at the center of the filming apparatus. Prior to the start of the trial, a single individual was placed into the acclimation cylinder for 5 min. The cylinder was then removed, and a camera (brand: Logitech, Lausanne, Switzerland; C920; resolution: 720 × 1280) was used to begin recording for a duration of 20 min. The recording duration was determined by preliminary experiments. After each trial, the water and all chironomid larvae were replaced, and the interior of the filming apparatus was thoroughly cleaned. Foraging behavior parameters were analyzed using EthoVision XT 9 software (EthoVision XT 9, Nodus, Wageningen, The Netherlands). The distance traveled and the time taken by an individual to locate and consume the first chironomid larva were defined as the foraging distance (cm) and foraging time (s), respectively. The ratio of foraging distance to foraging time was defined as the absolute foraging speed (cm·s^−1^), and its value divided by the individual’s body length was defined as the relative foraging speed (bl·s^−1^).

#### 2.3.4. Resting Metabolic Rate

Following the different starvation periods, 10 individuals were randomly selected from each group for the measurement of resting metabolic rate. The metabolic rate of each individual was measured using a custom-built “recirculating closed respirometer” [60]. The measurement method is briefly described as follows: Prior to the resting metabolic rate measurement, each individual was placed separately in a respiratory chamber of the recirculating closed respirometer for a 24 h acclimation period. Subsequently, the oxygen consumption rate of each individual was measured for 10 min periods at 8:00, 14:00, and 20:00. During the measurement, a dissolved oxygen meter (HQ_30_, Hach Company, Loveland, CO, USA) was used to record the dissolved oxygen level in the experimental respiratory chamber containing the individual every 10 s. The oxygen consumption rate for an individual was calculated from the slope of the dissolved oxygen values (6 readings) within the respiratory chamber over 1 min as a function of time. Concurrently, the dissolved oxygen level in an empty respiratory chamber (to account for bacterial oxygen consumption) was measured for 10 min, and the slope of bacterial oxygen consumption at that time point was calculated using the same method. The average of all oxygen consumption rates (30 readings) measured for an individual over the day was taken as its resting metabolic rate (mg O_2_·kg^−1^·h^−1^). The resting metabolic rate of the individuals was calculated using the following formula [60]:

*M*O_2_ = (*S*_t_ − *S*_0_) × *V* × 60/(*M* × 1000) (4)

where *MO*_2_ (mg O_2_·kg^−1^·h^−1^) is the metabolic rate of the individual, *S*_t_ is the slope of the dissolved oxygen change over time in the experimental respiratory chamber during the 1 min period, *S*_0_ is the slope of the dissolved oxygen change in the blank chamber (bacterial oxygen consumption), *V* is the volume of the respirometer (0.15 L), 60 is 60 min, and *M* is the body mass (g) of the individual.

#### 2.3.5. Hypoxia Tolerance

Following the different starvation periods, 24 individuals were randomly selected from each group and divided into 4 replicates (6 individuals per replicate) for the measurement of hypoxia tolerance. The method for determining the hypoxia tolerance parameters *ASR*_100_ and *LOE*_100_ is briefly described as follows [40]: A recirculating pump was placed at the bottom of a custom-built cylindrical hypoxia tolerance testing apparatus (50 L) to create internal water circulation, ensuring uniform dissolved oxygen levels throughout the apparatus. The water surface was sealed with a transparent plastic film. Prior to the test, six independent wire mesh cages (length × width × height: 30 cm × 20 cm × 10 cm) were placed below the waterline, and one individual was placed into each cage for a 1 h acclimation period. Subsequently, nitrogen was introduced into the apparatus to rapidly reduce the dissolved oxygen level from normoxic conditions (approximately saturation) to 5 mg·L^−1^ within 3 min, and then further reduced to 2.5 mg·L^−1^ within the next 3 min. The dissolved oxygen level was maintained at each of these two levels for 0.5 h. Thereafter, the dissolved oxygen level was decreased in a stepwise manner in decrements of 0.1 mg·L^−1^. At this stage, each change in dissolved oxygen level was accomplished in less than 1 min and maintained for 0.5 h. Dissolved oxygen levels were continuously monitored throughout the experiment using a dissolved oxygen meter (HQ_30_, Hach Company). The dissolved oxygen level at which the last individual in a replicate continuously and vigorously surfaced to hit the plastic film for 5 s was recorded as the *ASR*_100_. The dissolved oxygen level at which the last individual in a replicate exhibited a ventral-side-up posture at the bottom of the mesh compartment was recorded as the *LOE*_100_.

### 2.4. Statistical Analysis

Data processing and statistical analyses were performed using Excel 2003 and SPSS 17.0 software. All data were tested for normality (Shapiro–Wilk test) and homogeneity of variances. If these assumptions were met, one-way analysis of variance (ANOVA) was used to examine the effects of starvation on parameters related to growth characteristics and locomotor performance. Analysis of covariance (ANCOVA), with body length or body mass as a covariate, was employed to test the effects of starvation on parameters related to foraging behavior, resting metabolic rate, and hypoxia tolerance. When significant differences were detected, multiple comparisons were conducted using the least significant difference (LSD) test. The effects of starvation on initial and final body mass, body length, and condition factor within the same starvation time treatment group were analyzed using a *T*-test. All data are presented as the mean ± standard error (mean ± SE). A probability (*p*) value of less than 0.05 was considered statistically significant.

## 3. Results

### 3.1. Growth Characteristics

The final body mass and final condition factor of each starvation group (S1, S2, and S4) were significantly lower than their respective initial body mass and initial condition factor (*p* < 0.05). The final body mass (*F*_2, 161_ = 4.412; *p =* 0.014) and final condition factor (*F*_2, 161_ = 4.107; *p =* 0.018) of the S4 group were significantly lower than those of the S1 and S2 groups. The final body length of the S4 group was significantly lower than its initial body length (*p* < 0.05). No significant differences in final body length were found among the three starvation groups. The daily change rate of body mass in the S1 group was significantly greater than that in the S2 and S4 groups (*F*_2, 161_ = 57.068; *p <* 0.001). No significant differences were observed in the daily change rate of body length among the three starvation groups (Table 1).

### 3.2. Locomotor Performance

Different starvation durations did not significantly affect the relative induction velocity (*F*_3,39_ = 2.599, *p* = 0.067) or the relative burst swimming speed (*F*_3,39_ = 2.361, *p* = 0.088) of the *A. davidianus* larvae. There were no significant differences among treatment groups in relative induction velocity or relative burst swimming speed (Table 2).

### 3.3. Foraging Behavior

Starvation had no significant effects on the total prey searching distance (*F*_3,39_ = 1.612, *p* = 0.204), relative prey searching speed (*F*_3,39_ = 0.575, *p* = 0.635), or prey searching time (*F*_3,39_ = 1.196, *p* = 0.326) of the *A. davidianus* larvae. No significant differences were observed among treatment groups in total prey searching distance, relative prey searching speed, or prey searching time (Table 2).

### 3.4. Resting Metabolic Rate

**Figure 1:**
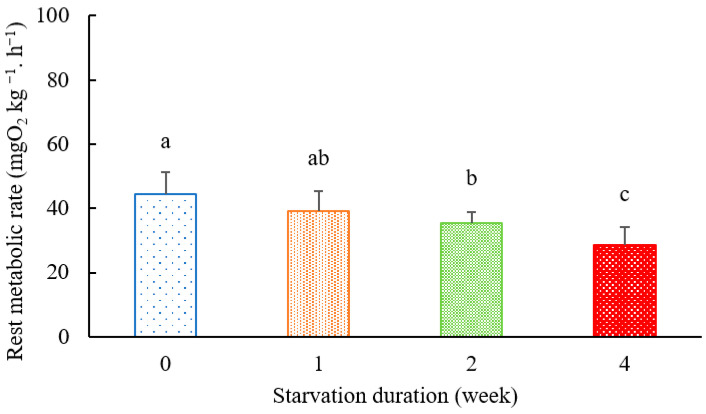
Effects of starvation on the rest metabolic rate in *A. davidianus* larvae (mean ± SE). Different lowercase letters indicate a significant difference (*p* < 0.05).

With prolonged starvation duration, the resting metabolic rate of the *A. davidianus* larvae decreased significantly (*F*_3,39_ = 11.833, *p* < 0.001). The resting metabolic rates of the S0, S1, S2, and S4 groups were 44.34, 39.32, 35.41, and 28.56 mg O_2_·kg^−1^·h^−1^, respectively. The resting metabolic rate was highest in the S0 group, intermediate in the S2 group, and lowest in the S4 group (Figure 1).

### 3.5. Hypoxia Tolerance

**Figure 2:**
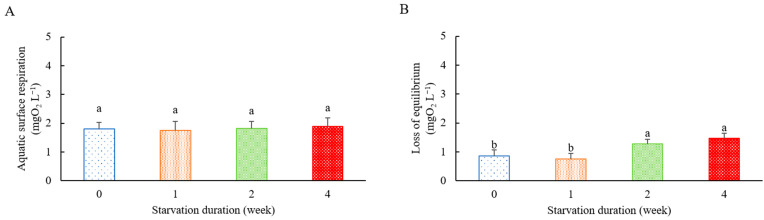
Effects of starvation on the hypoxia tolerance in *A. davidianus* larvae (mean ± SE). (**A**) aquatic surface respiration, *ASR*_100_; (**B**) loss of equilibrium, *LOE*_100_. Different lowercase letters indicate a significant difference (*p* < 0.05).

The aquatic surface respiration (*ASR*_100_) values of the S0, S1, S2, and S4 groups were 1.80, 1.75, 1.82, and 1.89 mg O_2_ L^−1^, respectively. Starvation had no significant effect on the *ASR*_100_ of *A. davidianus* larvae (*F*_3,15_ = 0.789, *p* = 0.525) (Figure 2A). No significant difference was found in the loss of equilibrium (*LOE*_100_) between the S0 and S1 groups (0.86, and 0.76 mgO_2_ L^−1^, respectively); however, the *LOE*_100_ values for both the S0 and S1 groups were significantly lower than those of the S2 and S4 groups (1.27, and 1.46 mgO_2_ L^−1^, respectively) (*F*_3,15_ = 7.339, *p* = 0.006) (Figure 2B).

## 4. Discussion

### 4.1. Effects of Starvation on Growth Characteristics of A. davidianus Larvae

In the absence of food supplementation, animals facing starvation stress must rely on the consumption of internal energy reserves to sustain basic physiological activities, which subsequently exerts a negative impact on their growth [1,2,65]. Previous studies have found that the effect of starvation on amphibian growth may be related to the species’ starvation tolerance and the duration of starvation [3,9,66]. Two weeks of starvation did not significantly affect the body mass and body length of Asiatic toads (*Bufo gargarizans*), Chinese fire-bellied newts (*Cynops orientalis*), and Andean toads (*Bufo spinulosus*) [67,68,69]. Starvation resulted in a significant decrease in body mass in African clawed frogs (*Xenopus laevis*) (starved for 3 and 7 months), Macedonian crested newts (*Triturus macedonicus*) and Buresch’s crested newts (*Triturus ivanbureschi*) (starved for 2 weeks), three-toed amphiumas (*Amphiuma tridactylum*) (starved for 3 and 6 months), toads (*Bombina maxima*) (starved for 60 days) and yellow belly toad (*Bombina variegata*) (starved for 2 weeks) [3,9,66,70,71].

*A. davidianus* larvae may face the threat of starvation in the wild, particularly during the overwintering season or when disturbances such as water quality deterioration and human activities induce stress responses. The present study found that 1, 2, and 4 weeks of starvation stress led to significant reductions in the body mass of Chinese giant salamander larvae by 11%, 12%, and 19%, respectively, but only resulted in body length decreases of 0.9%, 1.5%, and 2.8%, respectively. In this study, we did not set up parallel control groups corresponding to each starvation group (S1, S2, and S4) to monitor data on continuous growth under normal feeding conditions. The S0 group was not really a control group, because growth-related parameters were not measured in this group over the same time period as the other starvation groups. Therefore, the design of this control group has certain limitations. Due to the possibility that individuals in the parallel control groups might have continued to grow and gain mass during the study period, the actual negative effects of starvation on the body mass of *A. davidianus* larvae may have been more significant than the results observed in this experiment. These data indicate that the negative impact of starvation on body mass was significantly greater than that on body length in *A. davidianus* larvae. This finding is similar to the results of our previous study [4]. This phenomenon has also been observed in studies on other urodeles. For instance, starvation did not significantly affect the body length of Macedonian crested newts (*T. macedonicus*) and Buresch’s crested newts (*T. ivanbureschi*) (fasted for 2 weeks) [9], cave-dwelling salamanders (*Proteus anguinus*) (fasted for 18 months) [8], and three-toed amphiumas (*Amphiuma tridactylum*) (fasted for 1–6 months) [66]. Since the tail plays a crucial role in the locomotion of caudate amphibians, a relatively conservative change in body length may be more beneficial for these caudate amphibians to maintain locomotor performance under starvation stress, thereby enabling them to more effectively capture prey and evade predators.

The present study found that the final body mass of the 4-week starvation group was significantly lower than that of the 1-week and 2-week starvation groups. This indicates that starvation duration significantly affects the body mass of *A. davidianus* larvae. Similar results have been found in studies on other amphibians [3,66]. However, the daily change rate of body mass in the 1-week starvation group (−8.74% d^−1^) was significantly greater than that in the 2-week (−4.68% d^−1^) and 4-week (−3.72% d^−1^) starvation groups. This suggests that the negative impact of starvation on body mass was more pronounced during the early phase, which may be related to the relatively higher energy maintenance requirements during the initial period of starvation. A study found that larger individuals of a tropical amphibian (*Ceratophrys stolzmanni*) exhibited significantly higher activity levels, locomotor performance, and survival rates than smaller individuals [72]. Although our study did not investigate the reproductive behavior of adult *A. davidianus*, the poor physical condition of larvae after starvation may also affect their long-term adaptability and reproductive performance as adults, even if their physical condition can improve once food supply is restored. Therefore, further research on the reproductive capacity and survival rate of *A. davidianus* after starvation is needed in the future.

### 4.2. Effects of Starvation on Locomotor Performance of A. davidianus Larvae

Induction velocity primarily reflects the ability of aquatic animals to perceive changes in water flow. Due to its significant interspecific variation, it holds important reference value in the conservation practices of aquatic animals [73,74]. Previous studies on induction velocity have mainly focused on fish [15,16]. In the present study, the relative induction velocity observed in *A. davidianus* larvae was generally consistent with data obtained in our previous studies on juvenile *A. davidianus* (approximately 2.8 bl s^−1^) [60,75]. Furthermore, 4 weeks of starvation stress did not significantly alter the relative induction velocity of *A. davidianus* larvae. Combined with our previous findings that anaerobic exercise training and feeding levels did not affect the induction velocity of juvenile *A. davidianus* [60,75], the results of this study further suggest that the induction velocity in the early life stage of *A. davidianus* is relatively conservative and was not significantly affected by 4 weeks of starvation stress.

Amphibians cannot survive without an aquatic environment, and their swimming ability is crucial for behaviors such as foraging and predator avoidance [76,77,78]. This study found that the relative burst swimming speed of *A. davidianus* larvae in the control group was approximately 4.3 bl s^−1^, which is similar to the results of our previous studies on juvenile *A. davidianus* [60,75]. Moreover, although the resting metabolic rate decreased significantly, 1–4 weeks of starvation stress did not significantly affect the relative burst swimming speed of *A. davidianus* larvae. Since the locomotor performance of aquatic animals is influenced not only by their maintenance metabolism but also by physiological traits such as maximum locomotor metabolism and locomotor efficiency [11,39], the intrinsic reasons why the locomotor performance of *A. davidianus* larvae remains stable under starvation stress warrants further investigation. Previous studies have found that the swimming speed of brown tree frog tadpoles (*Litoria ewingii*) subjected to restricted feeding (half the normal ration) for 14 days was significantly reduced [20]. A blind cave-dwelling salamander (*Proteus anguinus*) and a surface-dwelling salamander (*Euproctus asper*) exhibited a pattern of initially increasing and then decreasing displacement speed during long-term starvation (240 days and 90 days, respectively) [21]. This indicates that there are significant interspecific differences in the locomotor performance of amphibians under food-scarce conditions. Similar to induction velocity, the burst swimming speed of *A. davidianus* larvae was also relatively conservative under starvation stress. This “maintenance” strategy of locomotor function may be beneficial for enhancing their ability to forage and avoid predators in environments with scarce food resources, thereby improving their survival fitness. However, although there was no statistically significant difference (*F*_3,39_ = 2.361, *p* = 0.088), the relative burst swimming speed of *A. davidianus* larvae showed a decreasing trend with prolonged starvation. In particular, four weeks of starvation resulted in a 12% decrease in the relative burst swimming speed. Therefore, the negative impact of long-term food deprivation on the locomotor performance of this species should be further addressed in future studies.

### 4.3. Effects of Starvation on Foraging Behavior of A. davidianus Larvae

Throughout the long process of evolution, an animal’s foraging strategy is closely related to its living environment [79]. Changes in the nutritional status of amphibians due to food restriction can significantly impact their foraging behavior [33,34]. For example, 5 days of starvation significantly shortened the attack latency of graybelly salamanders (*Eurycea multiplicata*), but did not significantly change their prey handling time [33]. Four days of starvation stress not only increased the activity level of pool frog (*Rana lessonae*) and edible frog (*Rana esculenta*) tadpoles, but also extended the duration of their foraging activity [34]. The present study found that 1–4 weeks of starvation stress had no significant effects on the total foraging distance, relative foraging speed, or foraging time of *A. davidianus* larvae. This indicates that there are interspecific differences in the foraging behavioral responses of amphibians when facing starvation stress. The foraging behavior characteristics of *A. davidianus* larvae were also relatively conservative during food restriction, which may be related to the “maintenance” strategy observed in their locomotor performance (burst swimming speed). Such a behavioral strategy may be beneficial for maintaining foraging ability in food-scarce environments, thereby increasing their probability of surviving periods of starvation stress.

### 4.4. Effects of Starvation on Hypoxia Tolerance of A. davidianus Larvae

In natural aquatic environments, animals are frequently subjected to varying degrees of hypoxic stress [80]. Metabolic suppression is often accompanied by a reduced demand for oxygen, which facilitates better adaptation to hypoxic conditions and is therefore considered a crucial mechanism for animal survival in hypoxic environments [43,80]. For example, Sichuan sinibrama (*Sinibrama taeniatus*) exhibited significantly enhanced hypoxia tolerance after 28 days of starvation stress, which researchers attributed to a potential decrease in maintenance metabolic rate during starvation [45]. In contrast, 16 days of starvation did not significantly alter the resting metabolic rate or hypoxia tolerance of largemouth bass (*Micropterus salmoides*) [46]. In the present study, with prolonged starvation duration (2–4 weeks), the resting metabolic rate of *A. davidianus* larvae decreased significantly, indicating that they cope with starvation stress by reducing maintenance energy expenditure. The average body length of the experimental subjects in this study was approximately 9.78 cm, yet they were housed individually in cages measuring 10 cm in length, width, and height. This spatial constraint may have had an inhibitory effect on their metabolic rate. Therefore, the reduction in maintenance metabolic energy observed in *A. davidianus* larvae may have been influenced, to some extent, by the limited housing conditions provided to them. Starvation for 1–4 weeks did not result in significant differences in the *ASR*_100_ of *A. davidianus* larvae. However, 2–4 weeks of starvation led to a significant increase in their *LOE*_100_. These data suggest that while the critical hypoxia tolerance of *A. davidianus* larvae was not affected by starvation stress, their extreme hypoxia tolerance was impaired with prolonged starvation, which may subsequently negatively impact their survival. This impairment might be related to decreased oxygen uptake and transport capacity, as well as impaired anaerobic metabolic function during starvation [48,81,82]. This phenomenon has also been observed in studies on North African catfish (*Clarias gariepinus*) [48], and the underlying mechanisms warrant further investigation.

## 5. Conclusions

In summary, 1 to 4 weeks of starvation stress had a significant negative impact on the body mass of *A. davidianus* larvae, with this negative effect being more pronounced during the early phase of starvation. The body length of *A. davidianus* larvae remained relatively conservative under the same starvation stress conditions. The locomotor performance and foraging behavior of *A. davidianus* larvae were not affected by starvation (up to 4 weeks), which may be beneficial for maintaining foraging and predator avoidance abilities in food-scarce environments, thereby enhancing their survival fitness. However, with prolonged starvation duration (2–4 weeks), the extreme hypoxia tolerance of *A. davidianus* larvae was impaired, potentially due to excessive energy consumption and disruptions in physiological regulation under starvation stress. The control group (S0) was a separate pre-control group, and parallel control groups for each starvation group (S1, S2, and S4) at the same time points of starvation were not established in this study. Therefore, the negative effects of starvation on *A. davidianus* larvae may not have been fully revealed in this study. This limitation in the setup of the control group should be avoided in future studies. Furthermore, when *A. davidianus* larvae are exposed to multiple harsh environmental stressors in the wild, such as temperature fluctuations, hypoxia variability, intraspecific competition, and predation risk, the negative effects of starvation on their body mass and hypoxia tolerance may become more pronounced, potentially leading to carryover effects on subsequent life stages. Therefore, the issue of food availability should be fully considered in conservation practices such as the stock enhancement of *A. davidianus* larvae. Additionally, hypoxia tolerance warrants greater attention in future conservation research on this species. It is recommended to select a release site for *A. davidianus* larvae that offers relatively abundant prey and moderate water flow. This will help prevent the larvae from experiencing multiple stressors, such as food scarcity and low oxygen levels, thereby enhancing their post-release survival rate.

## Figures and Tables

**Table 1:** The effect of starvation on the growth in *Andrias davidianus* larvae (mean ± SE).

	Starvation Time (Week)				Significance
	Control	1	2	4	Starvation Effect
Sample (N)	54	54	54	54	--
Initial body mass (g)	5.46 ± 0.12 ^a^	5.42 ± 0.11 ^a^	5.35 ± 0.09 ^a^	5.42 ± 0.13 ^a^	*F*_3, 215_ = 0.157; *p* = 0.925
Initial body length (cm)	9.77 ± 0.07 ^a^	9.73 ± 0.08 ^a^	9.79 ± 0.06 ^a^	9.79 ± 0.08 ^a^	*F*_3, 215_ = 0.118; *p* = 0.949
Initial condition factor (100 × g cm^−3^)	0.58 ± 0.01 ^a^	0.58 ± 0.01 ^a^	0.57 ± 0.01 ^a^	0.57 ± 0.01 ^a^	*F*_3, 215_ = 1.295; *p* = 0.277
Final body mass (g)	--	4.81 ± 0.10 ^a^*	4.69 ± 0.09 ^a^*	4.39 ± 0.11 ^b^*	*F*_2, 161_ = 4.412; *p =* 0.014
Final body length (cm)	--	9.64 ± 0.07 ^a^	9.64 ± 0.06 ^a^	9.51 ± 0.08 ^a^*	*F*_2, 161_ = 1.012; *p =* 0.366
Final condition factor (100 × g cm^−3^)	--	0.53 ± 0.01 ^a^*	0.52 ± 0.01 ^a^*	0.50 ± 0.01 ^b^*	*F*_2, 161_ = 4.107; *p =* 0.018
Daily rate of body mass change (% d^−1^)	--	−8.74 ± 0.54 ^b^	−4.68 ± 0.26 ^a^	−3.72 ± 0.13 ^a^	*F*_2, 161_ = 57.068; *p <* 0.001
Daily rate of body length change (% d^−1^)	--	−1.46 ± 0.53 ^a^	−1.13 ± 0.25 ^a^	−1.00 ± 0.13 ^a^	*F*_2, 161_ = 0.453; *p =* 0.636

Values in each row with different lowercase letters indicate a significant difference (*p* < 0.05). The asterisk (*) denotes a significant difference between the initial and final values within the same treatment group (*p* < 0.05).

**Table 2:** The effect of starvation on several variables related to the locomotor performance, foraging behavior in *Andrias davidianus* larvae (mean ± SE).

	Starvation Time (Week)				Significance	
	Control	1	2	4	Covariate	Starvation
Sample (N)	10	10	10	10	--	--
Relative induction flow speed (bl s^−1^)	3.00 ± 0.48 ^a^	3.17 ± 0.62 ^a^	2.70 ± 0.56 ^a^	2.48 ± 0.57 ^a^	--	*F*_3, 39_ = 2.599, *p* = 0.067
Relative burst swimming speed (bl s^−1^)	4.30 ± 0.66 ^a^	4.56 ± 0.65 ^a^	4.08 ± 0.69 ^a^	3.78 ± 0.63 ^a^	--	*F*_3, 39_ = 2.361, *p* = 0.088
Total foraging distance (cm)	587.07 ± 328.44 ^a^	829.48 ± 541.63 ^a^	1324.38 ± 1083.18 ^a^	1062.24 ± 665.46 ^a^	*F*_1, 39_ = 6.397, *p* = 0.016	*F*_3, 39_ = 1.612, *p* = 0.204
Relative foraging speed (bl s^−1^)	0.16 ± 0.13 ^a^	0.17 ± 0.19 ^a^	0.23 ± 0.21 ^a^	0.14 ± 0.08 ^a^	*F*_1, 39_ = 1.922, *p* = 0.174	*F*_3, 39_ = 0.575, *p* = 0.635
Foraging time (s)	495.62 ± 304.76 ^a^	707.43 ± 463.55 ^a^	749.44 ± 413.00 ^a^	893.51 ± 410.63 ^a^	*F*_1, 39_ = 1.260, *p* = 0.269	*F*_3, 39_ = 1.196, *p* = 0.326

Values in the same row with the same superscript lowercase letters indicate no significant difference. bl, body length.

## Data Availability

The data presented in this study are available on request from the corresponding author. The data are not publicly available due to privacy/ethical restrictions.
